# Ticks - public health risks in urban green spaces

**DOI:** 10.1186/s12889-024-18540-8

**Published:** 2024-04-13

**Authors:** Thérese Janzén, Firoza Choudhury, Monica Hammer, Mona Petersson, Patrik Dinnétz

**Affiliations:** Department of Natural Science, Environment and Technology, Södertörn University, Hudding, Sweden

**Keywords:** Exposure, Habitat, Hazard, Human activity, *Ixodes persulcatus*, *Ixodes ricinus*, Microhabitat, Recreation, Tick-borne diseases

## Abstract

**Background:**

Urban green spaces are important for human health, but they may expose visitors to tick-borne diseases. This not only presents a potential public health challenge but also undermines the expected public health gains from urban green spaces. The aim of this study is to assess the public health risk of tick-borne diseases in an urban green space used for recreation in Stockholm, Sweden.

**Methods:**

We used a mixed method approach identifying both the magnitude of the tick hazard and the extent of the human exposure to tick-borne diseases. At six entry points to an urban green space, we sampled ticks and documented microhabitat conditions from five randomly assigned 2 m × 2 m plots. Surrounding habitat data was analyzed using geographical information system (GIS). Nymphs and adult ticks were tested for *Borrelia burgdorfer*i sensu lato and *Anaplasma phagocytophilum* using TaqMan qPCR*.* Positive *B*. *burgdorferi* (s.l.) ticks were further analyzed by nested PCR amplification and sequence analysis. Population census data and visitor count data were used to estimate the degree of human exposure to tick-borne diseases. To further understand the degree to which visitors get in contact with infected ticks we also conducted interviews with visitors to green spaces.

**Results:**

High tick densities were commonly found in humid broadleaved forest with low field vegetation. High pathogen prevalence was significantly correlated with increasing proportions of artificial areas. Integrating the tick hazard with human exposure we found that the public health risk of tick-borne diseases was moderate to high at most of the studied entry points. Many of the visitors frequently used urban green spaces. Walking was the most common activity, but visitors also engaged in activities with higher risk for tick encounters. Individual protective measures were connected to specific recreational activities such as picking berries or mushrooms.

**Conclusions:**

The number of visitors can be combined with tick inventory data and molecular analyses of pathogen prevalence to make crude estimations of the public health risk of tick-borne diseases in urban green spaces. The risk of encountering infected ticks is omnipresent during recreational activities in urban green spaces, highlighting the need for public health campaigns to reduce the risk of tick-borne diseases.

**Supplementary Information:**

The online version contains supplementary material available at 10.1186/s12889-024-18540-8.

## Background

Green spaces in urban environments provide health benefits to residents and conserve biodiversity and ecosystem functions [1]. However, urban green spaces can also increase the risk for contact between humans and animal-associated pathogens [2, 3]. Viable tick populations are increasing in many urban green spaces, and prevalence of tick-borne pathogens in urban settings are even comparable to similar habitats in rural areas [4–6]. This not only presents a potential public health challenge but can also undermine the public health gain from urban green spaces [7].

Urban green spaces include green infrastructures in and around cities used for recreational activities [8]. Urban green spaces can provide relief from the pressure of living in a city and are places where residents may be in touch with the natural cycle of the seasons and of wildlife [9]. Urban forests, parks, and open grasslands often form a network of dispersal corridors for wildlife migration [10, 11]. Wild ungulates can serve as feeding hosts for ticks bringing them close to urban areas, and small vertebrates such as rodents are important tick-borne pathogen reservoirs [12]. From a public health perspective, risks regarding tick-borne diseases should be studied in urban green spaces, where contact between infected ticks and humans may be frequent, due to the high human activity [13]. Still, the rates of contact with infected ticks in urban green spaces used for recreation remains inadequately quantified [14, 15].

In Europe, the geographical distribution of two medically important tick species, *Ixodes ricinus* and *I. persulcatus,* have expanded significantly during the last few decades [16, 17]*.* Lyme Borreliosis (LB), the most frequently reported tick-borne disease, is caused by spirochetes from the *Borrelia burgdorferi* sensu lato (s.l.) complex [18]. These spirochetes are susceptible to several classes of antibiotics, and treatment of patients in an early stage of the disease usually results in complete recovery [19]. Still, severe outcome and long-term symptoms occur, burdening patients and healthcare systems. To mitigate disease burden, vaccines against LB are in development [20]. *B. miyamotoi*, which is clustered in the relapsing fever *Borrelia* group, is considered an emerging tick-borne disease [21]. *Anaplasma phagocytophilum,* the causative agent of Human Granulocytic Anaplasmosis (HGA), a febrile disease in humans, is the most widespread tick-borne disease in animals in the Northern Hemisphere causing significant economic losses [22]. In many European countries the bacterial tick-borne diseases are not notifiable, thus, it is difficult to determine the actual burden of these diseases [23].

Tick-borne disease risk has often been assessed by estimations of infected ticks in a certain area [24, 25]. However, these studies only measure the acarological risk and do not consider the complexity of human behavior and movement patterns, which is required for human-tick contact to occur [26]. In addition, the popularity of outdoor recreational activities among Europeans has resulted in an increased risk of tick-borne diseases [27]. Quantifying visits to green spaces by residents, and to understand the factors influencing their visits, remains a daunting task [28]. Therefore, a fundamental challenge in understanding the public health risk of tick-borne diseases, is the high heterogeneity in visitor use intensity and behavior in urban green spaces [29].

Proper risk assessment for tick-borne diseases includes a combination of factors related to the identification of the tick hazard and the characterization of human exposure [30]. The hazard is the number of infected ticks in the environment determined by the habitat conditions allowing ticks, hosts, and pathogens to complete their life cycles and to overlap [31]. Exposure is the spatial overlap between the hazard and humans, specifically the degree to which individuals get into contact with tick-infected areas [32]. The absence of a human vaccine for bacterial tick-borne pathogens has led to a focus on individual preventative measures to reduce the risk of tick-borne diseases [33]. Common public health recommendations include avoiding tick habitats, regular tick checks when walking in tick endemic areas, and wearing protective clothing such as long sleeves and tucking the pants into the socks [34]. Although these methods may be effective for preventing tick bites, the adoption of these behaviors varies [35–39]. To the best of our knowledge, our study is one of the first to simultaneously evaluate both tick hazards and visitor use intensity and behaviors to assess the risk of tick-borne diseases.

The aim of this study is to assess the public health risk of tick-borne diseases in an urban green space used for recreation. By using data on hazard and exposure, we evaluated how the risk of tick-borne diseases is affected by habitat characteristics, human usage, and individual behaviors. To assess the public health risk, data on hazard and exposure were combined into an index estimating the potential risk of tick-borne diseases. Our results can provide a basis for optimizing tick bite prevention and outreach measures to protect public health in urban green spaces.

## Methods

### Field sites

**Fig. 1:**
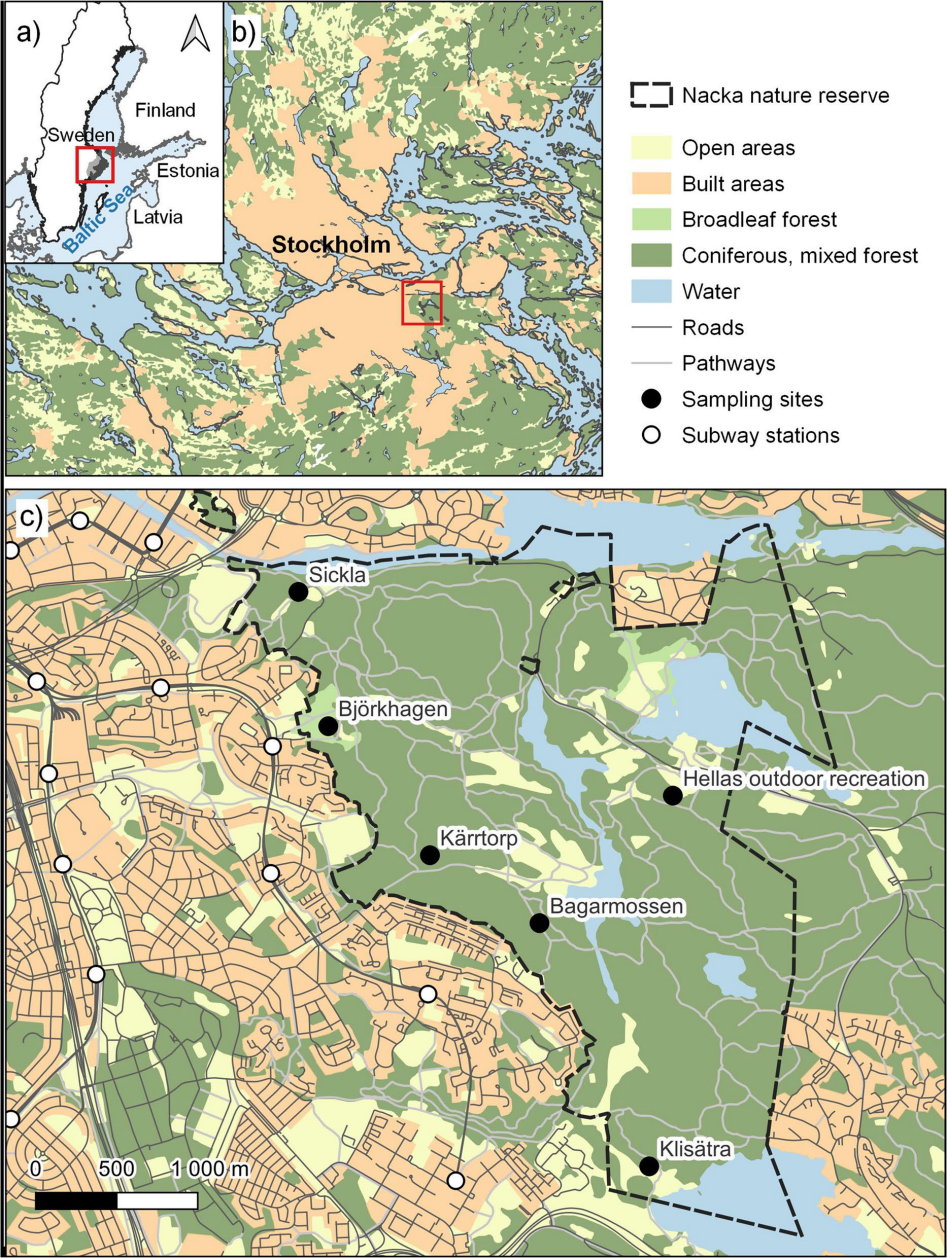
Location of the study area in Sweden (**a**) and Stockholm (**b**), and positions of the 6 sampling sites in Nacka nature reserve (**c**)

The Nacka nature reserve (Fig. 1) is located at the edge of the metropolitan area of Stockholm. It is one of Sweden’s most visited greenspace with more than 1,5 million visitors per year. The reserve is one of Stockholm’s largest, with approximately 730 hectares of forests, seminatural grasslands and lakes with several popular entry points, all close to the central parts of Stockholm. Forests and seminatural grasslands were previously used as pastures, fields, and meadows and the reserve is now a remnant of the old agricultural landscape. Several of the formerly open areas in the reserve are still kept open by mowing. During the last 70 years, this green space has mainly been used for recreational activities and offers opportunities for several outdoor activities such as walking, trail running, swimming, orienteering, biking, golf and picknick. The popular 1000 km long Sörmlands hiking trail starts at one of the entry points and runs through the southern part of the nature reserve.

### Public health risk assessment design

To assess the public health risk of tick-borne diseases in urban greenspaces, we used a mixed model approach identifying both the tick hazard, and the human exposure to ticks and tick-borne pathogens. To keep track of the daily visitors to the nature reserve, the municipality of Stockholm has placed six public count boxes at the entry points: Sickla, Björkhagen, Kärrtorp, Bagarmissen, Klisätra and Hellas recreations center (Fig. 1). The location of these public count boxes served as starting points for our data collection. Most entry points are easily reached, located close to the Stockholm subway system and public buses. Descriptions of all entry point is provided in Additional file 1.

To collect hazard data, we sampled ticks at the six entry points to establish tick densities and prevalence of bacterial pathogens. Tick-borne encephalitis (TBE) is also a public health problem in this area. However, the low prevalence of TBE-virus in ticks [40], makes TBEV a challenge to include in a risk assessment based on pathogen prevalence in ticks. Therefore, TBE was not included in this study. At all sampling sites, we documented microhabitat conditions that may support ticks and tick-borne bacterial pathogens. Large scale habitat features were analyzed using geographical information system (GIS). To collect exposure data, we obtained both residential census data from residential areas surrounding each entry point, and visitor data information from the public count boxes. To identify risk areas for tick-borne diseases in the urban green space, tick hazard and human exposures were integrated into a public health risk scale for each entry point. In addition, we also conducted interviews with visitors to the urban green space. Information from individual respondents provided details on important factors affecting risk of exposure to ticks and tick-borne diseases.

### Tick hazard data collection

In the summer of 2020, we collected ticks and field data in the areas surrounding the six public count boxes in the nature reserve. Each of the sites was visited twice, once in the beginning of the summer, and once at the end of the summer. Two different sites were visited during the same day, always between 10.00 am and 6.00 pm, and only on days without long-lasting precipitation. Since ticks and field data were collected in the areas surrounding the public count boxes, the site selection was not random. However, at each site, five randomly selected 2 m × 2 m sampling plots were selected. The random plot selection started from the public count boxes. The location of the first sampling plot was determined by holding a compass upside down and randomly rotating the graduation ring to first select a distance between 1 m – 36 m, and next a random compass direction between 0° – 360°. For distance, 10° on the graduation ring equals 1 m. The first sampling plot was used as starting point for selecting the second plot, and so on up to five plots. In total, 60 plots (5 plots × 6 sites × 2 sampling periods) were inventoried for ticks and field data. The coordinates of each random sampling plot were determined with a GPS hand receiver (Garmin eTrex 30).

### Tick collection

In Northern Europe*, I. ricinus* is active from spring to fall with adult tick activity peaking in July – August, and nymph activity peaking in September [41]. The seasonal activity of *I. persulcatus* is unimodal with activities in May – July, with the highest activity peak in May [42, 43]. Tick sampling was carried out in June, August and September which covers activity seasons for both tick species and encompassed a large portion of their highest activity periods. *Ixodes* species have three life cycle stages including larva, nymph, and adult ticks. In the case of *I. persulcatus*, the adult female is the primary stage that attacks humans [43]. For *I. ricinus* all three stages attack humans, but particularly the nymphs, which are the medically most important tick stage [44]. For this study, all tick stages were sampled to determine the overall tick density in urban green spaces.

Ticks were collected with the mopping technique [45, 46]. Briefly, we used a floor mop with a 0.7 × 0.7 m flannel cloth attached to the head of the mop (Additional file 2). In contrast to the common dragging technique, the mop is held in front of the user, and is moved anteriorly over the vegetation. The mop handle permits easy adjustments to different vegetation heights [46]. To estimate the tick density at each site, we inventoried the 5 random 2 m × 2 m plots. To increase the validity of estimates of pathogen prevalence at each site, we sampled more nymphs and adult ticks from the vegetation surrounding the five random plots. The surrounding vegetation was inventoried in random directions measuring the mopping time with an accuracy of 1 s. Total mopping time varied between 2 to 3 min for all sites. Using the average distance walked per second for the user and the width of the sampling blanket we calculated the total area covered, which ranged from 700m^2^ – 1000m^2^, varying with the landscape features at each site. All questing ticks attaching to the blanket were collected with tweezers, put into tubes, categorized according to life stage, and stored at -80ºC. Tick life stage categorization was later confirmed in the laboratory.

### Microhabitat data collection

**Fig. 2:**
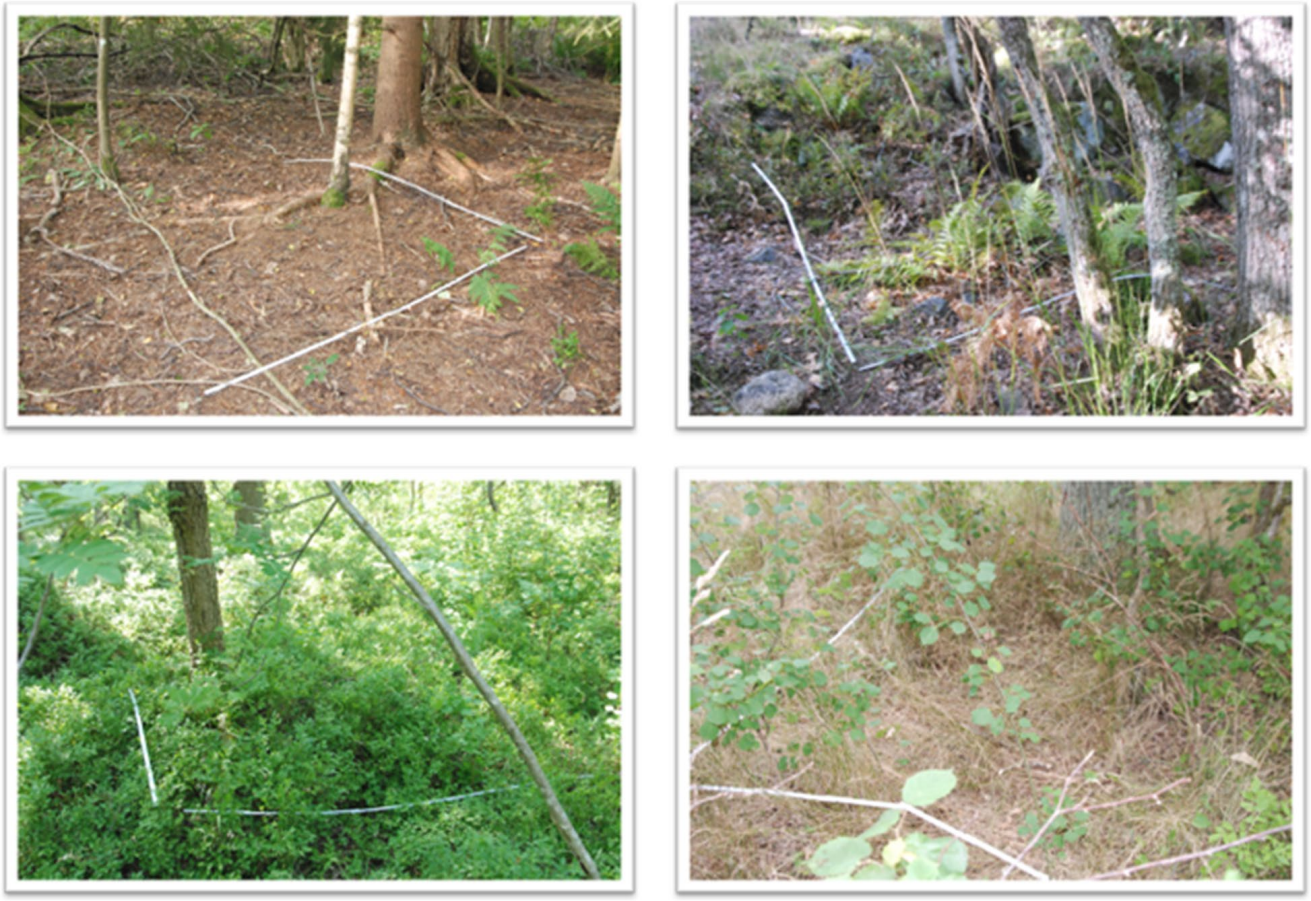
Examples of photo-documented sampling plots. The proportion of different field layer categories was used to determine microhabitat conditions

Microhabitat data was recorded for each 2 m × 2 m sampling plot. The microhabitat conditions included current air *Temperature*, average *Vegetation height* and *General field layer* in the sampling plots, as well as the *Tree stem density* surrounding each sampling plot. *Tree stem density* was estimated using the Bitterlich sampling technique [47]. To assess the *General field layer* composition and identify plant species, all 60 sampling plots were photo documented (Fig. 2). These photos were later processed by creating a 16 square grid layer in Power Point to determine the proportion of the *General field layer* including different plant species in each sampling plot. The *General field layer* categories were *Grass and herbs, Blueberry bushes, Dry vegetation* (heather and lichen)*, Shrubs, Leaves,* and *Bare ground.*

### Land cover data from GIS

For characterization of the surrounding habitat, we calculated a median coordinate for each sampling site from the geocoded positions of all five 2 m × 2 m plots. For each median coordinate, a 100 m buffer zone surrounding the sampling site was created and incorporated within a GIS layer together with pre-existing land cover maps. We used the 2018 National Land Cover Database of Sweden (Svenska marktäckedata, SMD), produced by the Swedish Environmental Protection Agency. These maps have a spatial resolution of 10 m and include six main land cover classes 1) forest and seminatural areas, 2) open areas, 3) arable land, 4) wetlands, 5) artificial areas and 6) lakes and oceans (Swedish land cover nomenclature available in Additional file 3). The main categories are further divided into subcategories with detailed information regarding the different land cover classes. To locate risk habitats based on the biological and ecological requirements for ticks and their associated pathogens, we extracted data on the main land cover classes with the exception for forest and seminatural areas where we included all ten different forest types present in the reserve. Of the ten different forest types, seven forest types were not on wetland; *Spruce forest, Pine forest, Mixed coniferous forest, Mixed forest, Broadleaved forest, Broadleaved hardwood forest* and *Broadleaved forest with broadleaved hardwood forest*, and three types were on wetland; *Mixed coniferous forest, Mixed forest* and *Broadleaved forest.* The nomenclature for Swedish land cover includes nonintuitive names with several definitions based on absence from certain features, like “not on wetland”. We have chosen to stick with the names in the nomenclature to allow for comparisons with other studies.

To calculate an urbanization index for each site, we used the proportion of *Artificial surfaces* in 1000 m buffer zones around the median coordinates for each site. Since Stockholm County is surrounded by water, we subtracted the proportion of *Open water* to adjust for presence of non-tick habitats (Eq. 1). The urbanization index ranges from 0 to 100 with the value 0 representing a completely natural or seminatural area and 100 representing a landscape with only artificial surfaces.

1 $$Urbanizationindex=Artificial\;surfaces\;\left(\text{\%}\right)/\left(100-Openwater\left(\text{\%}\right)\right)\times100$$

### Laboratory analyses

#### DNA extraction

DNA was extracted from the medically important tick stages of *I. ricinus* and *I. persulcaltus*, in total 132 nymphs and 5 adult ticks. DNA was extracted from each of the ticks using the Dneasy Blood & Tissue kit, following the supplementary protocol for detection of *Borrelia* DNA (QIAGEN, Chatsworth, California, USA). Each tick was cut with sterile needles along the torso, placed in a 1,5 ml Eppendorf tube with PBS buffer and a 5-mm steel bead (Qiagen, Hilden, Germany) and homogenized in a Tissue Lyser II (Qiagen) for 30 s at 30 Hz. The concentration of DNA extracted from each individual tick was controlled with a nanodrop©.

#### Molecular identification of tick species

Tick species identification was confirmed by molecular identification using a species-specific duplex Taqman real-time PCR essay for *I. ricinus* and *I. persulcatus* [48]. Species-specific probes Iri-I2-P4 and Ipe-I2-P4 are designed to target these two tick species (Additional file 4). The reaction mixture consisted of a 20-µl reaction with 10 µl Maxima® Probe qPCR Master Mix (2X) (Thermo Fisher Scientific, Stockholm, Sweden), 0.4 µl of forward and reverse primers, 0.3 µl of the probe Iri -I2-P4, and 0.2 µl of probe Ipe-I2-P4, 6.7 µl RNase-free water and 2 µl DNA. As a positive control, cDNA positive for *I. persulcatus*, previously confirmed by sequencing was added to each run*.* The *I. persulcatus* DNA was kindly provided by Prof Thomas Jaenson (Department of Organismal Biology, Evolutionary Biology Centre, Uppsala University, Sweden) through Dr Peter Wilhelmsson (Department of Biomedical and Clinical Sciences, Division of Inflammation and Infection, Linköping University, Sweden). Each run also contained one samples of milliQ water and one with DNA from the three-spine stickleback (*Gasterosteus aculeatus*) as negative controls. The PCR reactions were performed on the qPCR system CFX96 from Bio-Rad, using an activation step at 95 ºC for 5 min, and 45 cycles of 95 ºC for 10 s and 60 ºC for 60 s.

#### Detection of *Borrelia burgdorferi* (sensu lato) and determination of species

Detection of *B. burgdorferi* (s.l.) was conducted using a TaqMan real-time PCR assay [49]. The primers Borrelia-F and Borrelia-R and the probe Borrelia-P are designed to target the *B. burgdorferi* 16S rRNA gene to amplify a 131-bp long amplicon (Additional file 4). The reaction mixture resulted in a 20-µl reaction consisting of 10 µl Maxima® Probe qPCR Master Mix (2X) (Thermo Fisher Scientific, Stockholm, Sweden), 0.4 µl of each primer and probe, 6.8 µl RNase-free water and 2 µl DNA as previously described. In each run, 2 µl of a positive control with DNA extracted from of *B. garinii* (0.27*10^8^ cells/ml) was added. *B. garinii* (strain Lu59) was kindly provided by Sven Bergström (Umeå University, Umeå, Sweden) through Dr Peter Wilhelmsson (Department of Biomedical and Clinical Sciences, Division of Inflammation and Infection, Linköping University, Linköping, Sweden.) Each run also contained one sample with 2 µl milliQ water and one with 2 µl DNA from the three-spine stickleback (*Gasterosteus aculeatus*) as negative controls. The PCR reactions were performed on the qPCR system CFX96 from Bio-Rad, using an activation step at 95 °C for 5 min, and 50 cycles of 95 °C for 10 s and 60 °C for 60 s.

To determine the *B. burgdorferi* (s.l.) species of the samples positive in the real-time PCR, a nested conventional PCR using primers targeting the intergenic spacer region (IGS) between *5* and *23S* rRNA genes (Additional file 4), was applied. Nucleotide sequencing of the nested PCR products to determine the species of *Borrelia* was performed by Macrogen Inc. (Amsterdam, The Netherlands). All sequences were confirmed by sequencing both strands. The chromatograms were edited and analyzed by using BioEdit Software v7.0 (Tom Hall, Ibis Therapeutics, Carlsbad, CA). The consensus sequences were examined using Basic Local Alignment Search Tool (BLAST). An additional file shows all the aligned sequences (available in Additional file 2).

#### Detection of *Borrelia miyamotoi*

Detection of *B. miyamotoi* was done using a species-specific TaqMan real-time PCR assay [50]. The primers Bm_F and Bm_R, and the probe Bm_P are designed to target the *B. miyamotoi* flagellin B gene (faB) to amplify a 156-bp long amplicon (Additional file 4). The 20-µl reaction mixture, was prepared with 10 µl Maxima® Probe qPCR Master Mix (2X) (Termo Fisher Scientifc), 0.4 µl of each primer (10 µM; Life Technologies; Additional file 4), 0.4 µl of probe (10 µM; Life Technologies; Table 1), 3.8 µl RNase-free water and 5 µl DNA. As a positive control, a synthetic plasmid containing the target sequence of the TaqMan real-time PCR assay was used. The plasmid contained the target sequence, spanning the nucleotides 510–665 of the *B. miyamotoi* flagellin (faB) gene (GenBank: KT932823), synthesized and cloned into Eurofins standard vector (Eurofins Genomics, Ebersberg, Germany). Each run also contained samples with 2 µl milliQwater and 2 µl DNA from the three-spine stickleback (*G. aculeatus*) as negative controls. The PCR reactions were performed on the qPCR system CFX96 from Bio-Rad, using an activation step at 95 °C for 10 min, and 45 cycles of 95 °C for 5 s and 60 °C for 35 s, and lastly one cycle of 37 °C for 20 s.

**Table 1:** Microhabitat and habitat effects on tick abundance. Microhabitat conditions in and around each plot were analyzed in generalized linear mixed models with Poisson distribution. The effects from habitat features retrieved from GIS were analyzed in generalized linear mixed models with negative binomial distribution of residuals

	**Larvae**	**Nymphs**
**Microhabitat (2 m × 2 m plot)**		
Vegetation height	-0,06^***^	-0,03^*^
Grass and herbs	0,03^**^	0,02^*^
Shrub	0,07^***^	
Dry vegetation		-0,16^*^
Leaf litter	0.02^***^	
Temperature		-0,35^**^
**Habitat (100 m radius)**		
Artificial surfaces	-17.04^*^	
Broadleaved forest on wetland		8.49^*^

Significance levels: ^*^P < 0.05; ^**^P < 0.01; ^***^P < 0.001

#### Detection of *Anaplasma phagocytophilum*

Detection of *A. phagocytophilum* was done using a TaqMan real-time PCR assay [51]. The primers ApF and ApR, and the probe ApM are designed to target the *A. phagocytophilum* citrate synthase gene (gltA) to amplify a 64-bp long amplicon (Additional file 4). A 25-µl mixture reaction was prepared and consisted of 12.5 µl Maxima® Probe qPCR Master Mix (2X) (Termo Fisher Scientifc), 1.5 µl of each primer and 0.375 µl of probe, 7.125 µl RNase-free water and 2 µl DNA. As a positive control, a synthetic plasmid containing the target sequence of the TaqMan real-time PCR assay was used. The plasmid contained the target sequence, spanning the nucleotides 304–420 of the *A. phagocytophilum* gltA gene (GenBank: AF304137), synthesized and cloned into pUC57 vector (GenScript, Piscataway, NJ, USA). Each run also contained samples with 2 µl milliQwater and 2 µl DNA from the three-spine stickleback (*G. aculeatus*) as negative controls. The PCR reactions were performed on the qPCR system CFX96 from Bio-Rad, using an activation step at 95 °C for 10 min, and 40 cycles of 95 °C for 15 s and 60 °C for 60 s.

#### Exposure data collections

To characterize human exposure to the tick hazard in urban green spaces, a cross-sectional study design was applied by utilizing both population census data from the residential areas surrounding each sampling site and visitor data from the public count boxes located at the entry point into the nature reserve. In addition, to study how visitors use urban green spaces and to understand the degree to which humans get in contact with infected ticks, we conducted interviews with visitors to the nature reserve.

#### Population exposure

To assess the magnitude of public exposure to the tick hazard, we extracted residential census data available from Statistics Sweden (Statistiska centralbyrån, SCB). Data on number of residents is available in raster format with a resolution of 1km^2^ in the open Geodata section at SCB. We used data for individual raster cells covering the sampling sites as an estimate for potential daily visitors and an aggregation of 3 × 3 raster cells to cover 9km^2^ around the sampling site adding information of potential weekend visitors to the nature reserve. Population density provides an estimate of the numbers of residents living next to green spaces inhabited by infected ticks, but not the activity of residents in the urban green space. Visitor count data, on the other hand, can provide accurate estimates of resident’s visits to areas that area frequently used for recreational activities. All residential data was analyzed together with the visitor count data to estimate public exposure to the estimated infected tick hazard.

#### Visitor data collection

The visitor data information from the six public count boxes was provided by the City of Stockholm. Data included information on visiting dates and average number of visitors for weekdays and weekends during the counting period January – June 2020. For Hellas recreation center only data during the period May – July 2020 was available.

#### Individual exposure

To estimate the risk due to ticks and tick-borne pathogens at individual-level exposure, visitors were interviewed on-site at the different entry points to the nature reserve. The survey included questions and about visitors’ motives, behaviors, and movements patterns in different green spaces and in addition, their knowledge about ticks (survey questions available in Additional file 5). This survey was granted exemption from ethical approval from the Swedish Ethical Review Authority since no personal information was collected as part of the study. Visitors under the age of 18 were not eligible to participate. All respondents were approached in the nature reserve. They were informed that the interviewers were working in a research project about ticks and tick-borne diseases in urban green spaces used for recreation and were asked for a consent to participate in a brief survey regarding their recreational activities and their knowledge of ticks and tick-borne diseases. In addition, all participants were informed that they could stop the survey at any point. Each interview was conducted face-to-face, consisted of 17 questions, and lasted approximately 5 min per interviewee. Finally, to assess the knowledge and awareness of ticks, visitors were asked to identify an *I. ricinus* nymphal tick from photos of one tick and two other arthropods.

#### Data analysis

Analyses were performed using R v. 4.2.2 [52]. Because of the hierarchical nature of the data with tick samples from 5 plots nested within a site replicated at two different sampling occasions, generalized linear mixed models (GLMM) were used to analyze the effects of environmental data. Site was included as a random factor to account for the correlative structure within site. The models were analyzed assuming Poisson distributed residuals using package lme4 [53]. We used a backward stepwise model selection process based on the Akaike’s Information Criterion (AIC) and selected the most informative models with the lowest AIC score. To ensure that no severe multicollinearity existed between the predictor variables, variance inflation factors (VIFs) were computed for each predictor variable. Because no VIF was larger than 5, we did not exclude any variables due to multicollinearity. Model residuals were checked using the DHARMa package [54].

#### Microhabitat conditions

To assess the relationships between tick abundance and microhabitat conditions, tick abundance was analyzed as a function of the field layer composition measured as the proportional representation of the microhabitat components present in each of the sixty 2 m × 2 m plots. The abundance of tick larvae and nymphs were analyzed in separate models. Each model also included the plot factor *Vegetation height*, the surrounding *Tree abundance* as well as the time factor *Month* as predictors. Adult ticks were not included in the statistical analyses due to small sample size. The relationship between pathogen prevalence and microhabitat conditions could not be statistically analyzed due to very few infected ticks sampled in the individual 2 m × 2 m plots.

#### Habitat features

To assess effects of the surrounding habitat conditions, tick abundance (ticks sampled in the individual plots and surrounding vegetation) was analyzed as a function of the land cover types present in a 100 m radius buffer using GIS. The proportions of the different land cover types were included as fixed predictors. The abundance of tick larvae and nymphs in each sampling plot and from the surrounding vegetation, were analyzed in separate models. Each model also included the plot factor *Vegetation height*, the surrounding *Tree abundance* as well as the time factor *Month* as predictors. The site Sickla was divided into two independent sites because of the large geographical distance between the two sampling occasions. The models were fitted with a negative binomial distribution using the glmer.nb function in the package Ime4.

The relationship between tick-borne pathogens and environmental factors at habitat level were analyzed with the same predictive variables as in the analyses of tick abundance. However, the response variables were binary representing the presence/absence of pathogens in each single tick. *B. burgdoferi* (s.l.) and *A. phagocytophilum* infections were analyzed in two separate GLMM models assuming binomial residual distribution.

#### Human exposure

Correlations among population size, visitor counts, tick densities and pathogen prevalence at the different entry points to the urban green space were analyzed using Pearson’s correlation coefficients. To describe the visitors’ demographics, behavior, and prevention practices, descriptive and inferential statistics were used on the survey data from the interviews. Probability of recreational activity preformed, or use of protective measures was modeled as a response (binomial error distribution) using GLMMs. Age and gender were used as predictors for all three responses, and recreational activity type was included as a predictor for the use of protective measures. Respondents were included as random factor to account for repeated answers concerning activities and protective measures from the same visitor. The probability of identifying a nymphal tick from two other arthropods was analyzed using Generalized Linear Models (GLM) with a binomial distribution to evaluate the answers [correct, wrong] using age and gender as predictors.

#### Potential risk of tick-borne diseases

The potential risk of tick-borne diseases at each entry point was estimated by combining the tick hazard and human exposure estimates to an ordinal value ranging from 1 to 3, where the highest value corresponds to the highest hazard or exposure risk and vice versa. For tick hazard we used the result from a systematic review synthesizing tick densities and pathogen prevalence in Europe from 115 articles [55]. Based on their description of current situation in Europe we set the cut-off values for nymph and adult tick densities per 100m^2^ to: 1 (low) < 4 ticks per 100m^2^, 2 (moderate) 4 – 10 ticks per 100m^2^, and 3 (high) > 10 ticks per 100m^2^; and the cut-off values for bacterial pathogen prevalence to: Low < 14%, Moderate 14% – 26%, and High > 26%. The same cut-off values were used for both *B. burgdorferi* (s.l.) and *A. phagocytophilum*. For exposure estimates to the tick hazard, intervals based on the total range of all visitors per day at the different entry points were set to: Low < 1500 visitors, Moderate 1500 – 2000 visitors, and High > 2000 visitors. Using the sum of all values for hazard and exposure, we estimated the risk to Low 4 – 6, Moderate 7 – 9, or High 10 – 12, for each entry point (details in Additional file 6).

## Results

### Tick hazards in urban green spaces

Ticks and tick-borne bacterial pathogens were present at all entry points to the Nacka nature reserve. In total we collected 479 questing ticks, 342 larvae, 132 nymphs and 5 adults. The density of questing nymphs and adult ticks varied from 0.8 to 4.8 per 100m^2^ among the different sites. All collected nymphs and adult ticks belonged to the species *I. ricinus*. Hence, we did not find any *I. persulcatus* in Nacka nature reserve. The overall *B. burgdorferi* (s.l.) prevalence was 26.7%, ranging from 16.2% to 47.6% among the different sites (Table 4). Of the 44 samples positive for *B. burgdorferi* (s.l.) using real-time PCR, we identified 29 (65%) *B. afzelii*, 1 (0.02%) *B. garinii*, 1 (0.02%) *B. bavariensis*, and 1 (0.02%) *B. burgdorferi* s.s. (Additional file 7). The rest of the samples were not identified to species level, and therefore categorized as *Borrelia* spp. None of the sampled ticks were infected with *B. miyamotoi*. The overall prevalence for *A. phagocytophilum* was 28.2% ranging from 7.9% to 58.3% among the different sites. Both *B. burgdorferi* (s.l.) and *A. phagocytophilum* were detected at all sampling sites. At the most urban site, only16 nymphs were collected increasing the risk for noticeable random deviations of prevalence data from low number effects on proportional representation of binomial outcomes. In a case like this, the fate of one single individual tick can add 6.25% to the prevalence of one of the pathogens. Therefore, we need to be cautious about the accuracy of the prevalence, but we can still conclude if the prevalence is low, moderate, or high.

### Microhabitat conditions

Local tick densities for both larvae and nymphs were significantly higher in sampling plots where the field layer was low or even absent, compared to sampling plots with high *Vegetation height* (Table 1). There was also a significantly positive effect of the proportion of *Grass and herbs* in the sampling plots (Table 1). The proportion of *shrubs* and *Leaf litter* in the sampling plots had a significant positive effect on local larva abundance (Table 1). *Dry vegetation* such as heather or lichen, and the ambient air temperature had significant negative effects on local nymph abundance (Table 1). In this study, ticks were collected in temperatures ranging from 11 – 27 °C, and most of the nymphs were collected in temperatures below 24 °C.

### Habitat features

The degree of urbanization ranged from 1.5% at Hellas recreation center to 42.8% at Sickla entry point. The proportion of *Artificial areas* in the surrounding habitat had a significant negative effect on the presence of tick larvae (Table 1). The proportion of *Broadleaved forests on wetland* surrounding the sampling site had a significant positive effect on the presences of tick nymphs (Table 1). For the prevalence of *B. burgdorferi* (*s.l.*) there were significant positive effects from the proportion of *Artificial surfaces* and *Inland water*, and negative effects from the proportion of *Open areas* (Table 2). The prevalence of *A. phagocytophilum* was significantly positively affected by the proportion of *Artificial surfaces* and *Mixed coniferous forest not on wetland* (Table 2).

**Table 2:** Estimated coefficients for the relationship between the number of ticks infected with bacterial tick-borne pathogens and regression factors of the proportional representation of different surrounding habitat types analyzed with generalized linear mixed models assuming binomial distribution of residuals

Habitat 100 m radius	*Borrelia burgdorferi* (s.l.)	*Anaplasma phagocytophilum*
*Artificial surfaces*	5.09^*^	4.55^*^
*Open areas*	-2.98^***^	
*Mixed coniferous not on wetland*		4.55^***^
*Inland water*	21.61^***^	

Significance levels: ^*^P < 0.05; ^**^P < 0.01; ^***^P < 0.001

### Exposure to ticks and tick-borne pathogens

**Fig. 3:**
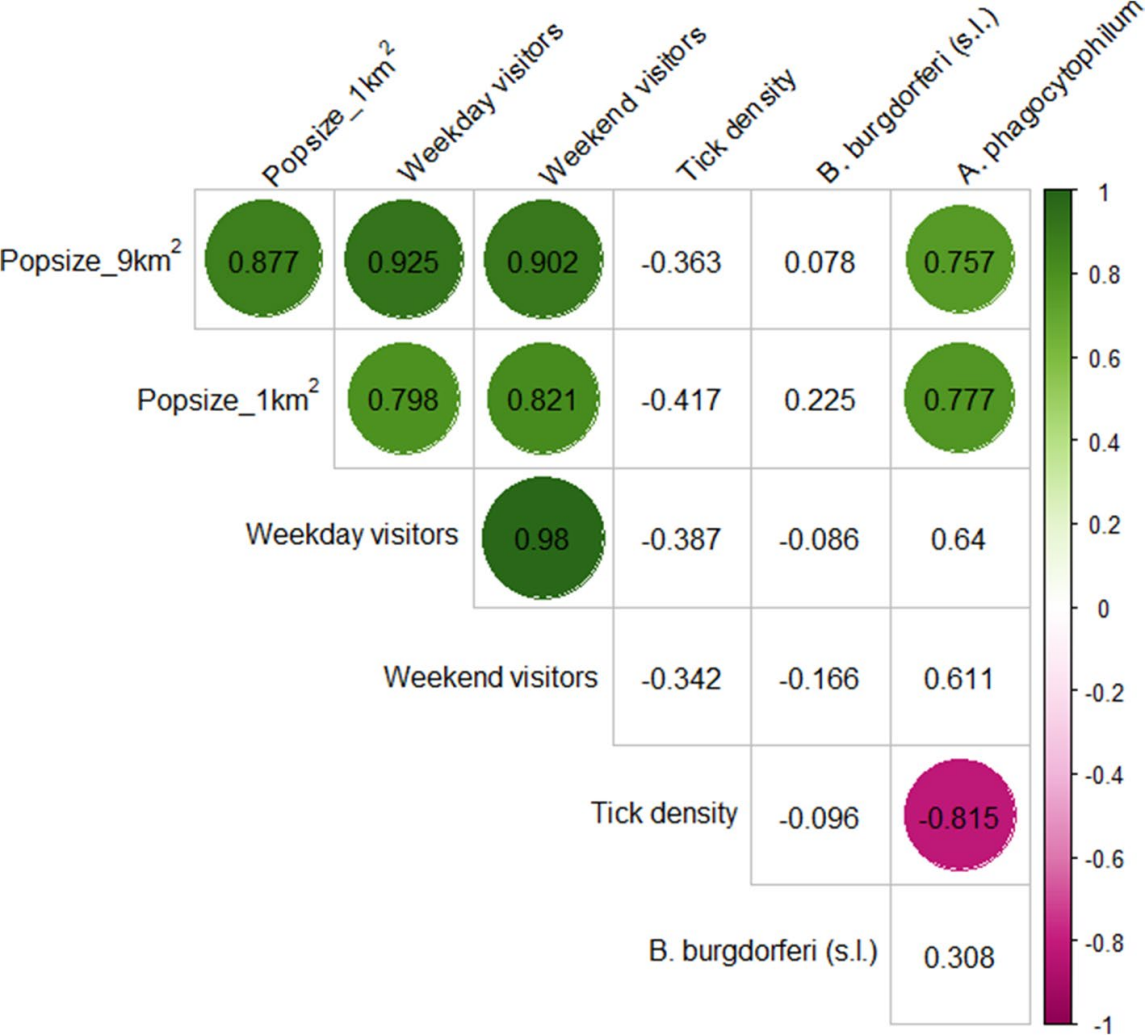
Correlations between human population size close to the entry point (9 km^2^) and very close to the entry point (1 km^2^), and number of visitors, tick density and pathogen prevalence. The color shows the strength of the correlation with green color indicating a strong positive correlation and pink color, a strong negative correlation. Absence of color indicate a non-significant correlation (*p* > 0.05). In this correlation matrix Sickla entry point was divided into two independent sites as in the habitat models

Exposure data represents the potential intensity of contact that people have with ticks and tick-borne pathogens. The entry points Sickla, Kärrtorp and Björkhagen, all close to highly urbanized areas had large human populations living in close proximity (Table 3 and Fig. 3). There were also more visitors entering the nature reserve at these points (Fig. 3). The least urban site, Hellas recreation center, had low tick density but high prevalence of tick-borne pathogens (Table 3). The intermediate urban sites Bagarmossen and Klisätra had high tick densities but low prevalence of tick-borne pathogens. Prevalence of *A. phagocytophilum* is significantly negatively correlated to tick density, meaning that there can be a high prevalence of *A. phagocytophilum* in areas with low tick density (Fig. 3). *A. phagocytophilum* infected ticks are also positively correlated to human population densities, and high prevalence were found in the most urbanized areas of the urban green space (Fig. 3).

**Table 3:** Population size as number of residents per 1km^2^, and 9km^2^, weekday and week-end visitors, tick abundance, tick density, *B. burgdorferi (s.l.)*, and *A. phagocytophilum* at all studied entry points to Nacka nature reserve

**Sites**	Population 1km^2^	Population 9km^2^	Weekday visitors	Week-end visitors	Total number of ticks	Tick density /100m^2^ (nymphs + adults)	*Borrelia burgdorferi* (s.l) %	*Anaplasma phagocytophilum* %
*Bagarmossen*	124	16121	370	636	85	4.8	18.4	7.9
*Björkhagen*	5747	48765	887	1363	97	4.6	35.9	20.5
*Hellas recreation center*	0	8159	371	582	26	1.5	47.6	28.6
*Klisätra*	969	22329	419	847	76	3.4	16.2	24.3
*Kärrtorp*	6739	28047	655	1157	101	1.9	17.6	29.4
*Sickla*	11563	82648	1024	1488	94	0.8	25.0	58.3

### Risk of tick-borne diseases in urban green spaces

**Fig. 4:**
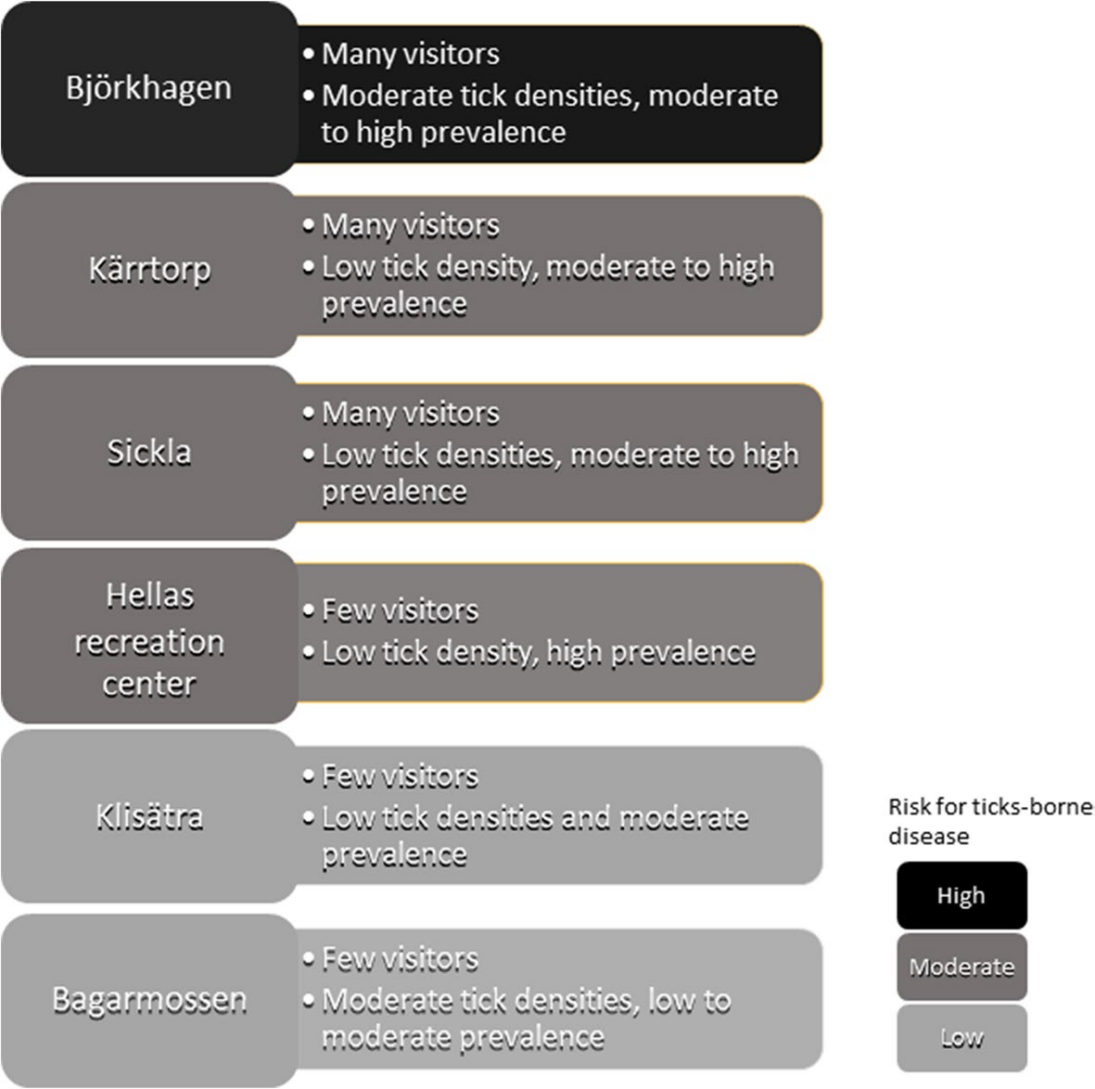
Risk of exposure to tick-borne pathogens at the differ entry points to Nacka nature reserve

When combining the tick hazard with human exposure estimated from number of visitors entering the nature reserve at each entry point, we found that the tick-borne disease risk was low at Klisätra and Bagarmossen, high at Björkhagen, and moderate at all other sites (Fig. 4). At Björkhagen entry point, many visitors enter the nature reserve, tick densities were high, and the prevalence of *B. burgdorferi* (s.l.) and of *A. phagocytophilum* were moderate to high (Fig. 4)*.*

### Behavior and movement patterns among visitors

A total of 102 visitors to the Nacka nature reserve were interviewed to gather information on sources of exposure to ticks and tick-borne pathogens and behavioral parameters effecting the risk. The study sample included 60 (59%) women and 42 (41%) men. Seventy-five percent of the respondents lived in close proximity (< 500 m) to the nature reserve and visited the area several times per week. However, 25% were visitors from other municipalities or even from other counties.

**Fig. 5:**
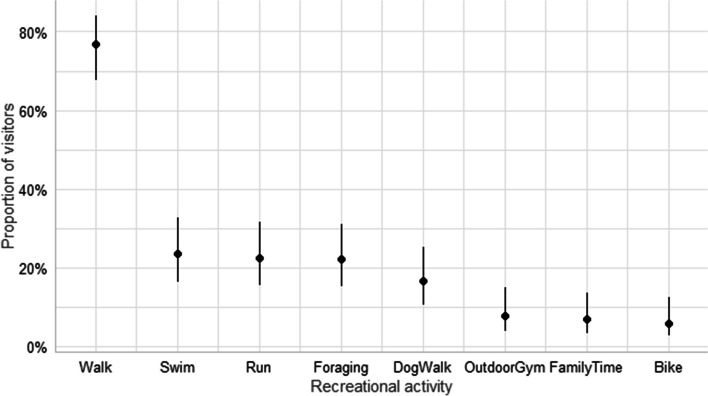
Activities performed by visitors in Nacka nature reserve. Foraging includes picking berries and mushrooms, and family time includes having a picknick or having family leisure time. Error bars indicate 95 percent confidence interval

Spending time outdoors was a common activity among the visitors and 77% reported that they spend time in urban green spaces several days per week. Walking was the most common activity, followed by swimming, running, and foraging the forest for berries or mushrooms (Fig. 5). When asked about their movement patterns, 85% of the visitors reported that they also walked off-trails when visiting green spaces.

Fifty-eight percent of the visitors reported that they have had several tick encounters during their life span, and 23% percent had several tick-bites every year. Twenty-two percent of the visitors reported having had a tick-borne disease (22 Lyme disease, 1 tick-borne encephalitis (TBE) infection). Among the visitors with a history of a tick-borne disease, 45% had several tick bites every year.

### Preventative behaviors

The results from our survey showed that the visitors used individual protective behaviors against ticks when spending time in typical tick habitats or when performing certain recreational activities. Several respondents tried to avoid tick bites by avoiding “typical tick habitat such as tall grass”, and by “performing tick checks” (Table 4). A less common strategy to prevent tick bites was to wear specific clothing including long pants or tucking pants into the socks (Table 4). Most visitor selected clothing depending on the weather or the recreational activity, and not as a strategy to avoid tick bites (Table 4). Protective measures against tick bites were mostly used during activities such as blueberry picking or mushroom hunting. Repellent was sometimes used (Table 4), but mostly with the intension to protect against mosquito bites.

**Table 4:** Prevention behaviors reported from visitors to urban green spaces. These protective measures were mostly used when performing certain recreational activities such as picking blueberries or mushrooms

Variables	Number (%)
Identify ticks (yes)	**76 (75%)**
Avoid tick habitat	**40 (39%)**
Regular checks	**40 (39%)**
Vaccinated (TBE)	**37 (36%)**
Repellent	**28 (27%)**
Clothing	**27 (26%)**

Three quarters of the visitors were able to identify a tick from other arthropods. For visitors with a history of a tick-borne disease, 100% could identify the tick. Among the visitors with a history of a tick-borne disease, 67% were women. There were no statistical differences between gender, or by different age groups, for the type of recreational activities performed or in usage of preventative measures.

## Discussion

This study aimed to assess the public health risk of tick-borne diseases in an urban green space used for recreation. Consistent with previous research [12, 55–57], we also found high tick densities and pathogen prevalence at most entry points, with tick densities decreasing with increasing degree of urbanization [58]. However, low tick density was also found at the least urban site, the Hellas recreation center, highlighting the influence of local properties and habitat types [59]. The Nacka nature reserve has several ecological niches with ideal tick habitats. Tick abundance was in our study area significantly associated with humid broadleaved forest. The highest tick densities were found at Björkhagen entry point, which is located in a lush Birch tree forest. Broadleaved forests provide a humid field layer, where the vegetation and litter layer protect ticks from desiccation during the off-host periods [60, 61]. Low tick densities were found in drier microhabitats, for example in areas with bare ground or were the forest floor consisted of large proportions of dry vegetation such as heather or lichen.

Ticks infected with *B. burgdorferi (s.l)* and *A. phagocytophilum* were found at all entry points, even in the highly urbanized parts of the green space. Pathogen prevalence is most likely determined by habitat conditions allowing ticks, hosts, and pathogens to complete their lifecycles and to overlap [62]. Stockholm County has a green infrastructure in the shape of a star, with protected green wedges that stretch from the surrounding rural areas all the way into the city. These wedges can serve as an ecological network for wildlife dispersal to urban green spaces. Large vertebrate hosts can utilize these networks bringing ticks to urban areas. Smaller vertebrates and birds inhabiting urban green spaces can be important tick-borne pathogen reservoirs [12]. Various tick host species have been identified as reservoirs for different *B. burgdorferi* genospecies [15]. In this study, the rodent-associated *Borrelia* genospecies *B. afzelii* was more prevalent than the bird-associated *B. garinii*. Knowledge of the prevalence of different *Borrelia* genospecies is important for public health, because different genospecies are associated with different clinical manifestations [63].

### Risk of tick-borne diseases in urban green spaces

Risk assessment of tick-borne diseases requires not only knowledge about the tick hazard, but also information about human exposure to infected ticks [64–67]. Models based on epidemiological data, such as tick-borne disease incidence, are advantageous in the way that disease cases unequivocally demonstrate human contact with the pathogen. However, there may also be a mismatch since diagnosed cases may in fact represent infections obtained elsewhere [15]. Since bacterial tick-borne diseases are not notifiable in Sweden, incidence data is not available. To estimate human contact rates with infected ticks, we used residential census data and visitor count data. The estimates of human activity varied between the different sites in the urban green space. Not so surprisingly, entry points located close to highly urbanized areas had the highest human population densities in the surrounding residential neighborhoods, as well as many entering visitors. Therefore, nearby residential population density data may be a good proxy for estimations of human exposure to tick-borne diseases in urban green spaces.

A high degree of urbanization can in itself be an important factor affecting exposure. Due to the high proportion of artificial surfaces in the surrounding landscape, there is a risk that individuals engaging in recreational activities underestimate the individual risk of being bitten by an infected tick [12]. The high prevalence of tick-borne pathogens, even at the most urbanized sites of the Nacka nature reserve, combined with the potential for underestimating individual risk, could lead to increased public health risks from tick-borne infections in urban green spaces. There will of course be differences in the strength and size of the relationship between residential population size and the number of visitors to different urban green spaces. We know that human communities in urban areas can be very diverse in their perception of nature [68], and that this can lead to large differences in how residents in different areas approach and use urban green spaces. Even if the residential areas around the Nacka nature reserve are heterogeneous and reflect a subset of the different residential areas found around a large urban center like the municipality of Stockholm, we still think that it is important to perform similar studies in other urban green areas in Stockholm as well as in other large cities.

All six entry points to the Nacka nature reserve except one, presented a moderate to high public health risk of tick-borne diseases. The highest risk was found at the Björkhagen entry point with high population size, many visitors, moderate density of ticks, and high prevalence of tick-borne pathogens. The least urban site, the Hellas recreation center, has no residential areas within 1km^2^ and rather few visitors during weekdays. During the weekends, visitors have more leisure time and come to enjoy the nature and a large variety of recreational activities. When recreational activities are performed in tick habitats and for longer periods of time, the possibility for human-tick contacts increases [15, 69]. If the prevalence of pathogens is high, which is the case at Hellas recreation center, the public health risk of tick-borne diseases will be high.

We do not seek to discourage residents from spending time in urban green spaces or participating in outdoor recreation. Instead, we want to identify risk factors for human exposure to infected ticks as a part of the process to prevent individuals from tick-borne diseases and reduce the public health burden associated with these illnesses. In the Northern Hemisphere, there is a longstanding tradition of protecting natural and semi-natural habitats in urban areas, primarily to facilitate recreational activities for urban residents. In several European cities, a maximum distance of 300 – 500 m to the nearest green space is used in planning processes of urban areas [8]. Given the evident risks of tick-borne diseases in urban green spaces, understanding individual residents’ behavior and movement patterns can be used to design tick bite prevention campaigns [69, 70].

### Outdoor activity patterns and prevention behaviors

Participating in outdoor recreational activities increase the risk of encountering infected ticks, especially in tick hazardous habitats [29], a concern exacerbated by the rising popularity of such activities among Europeans [27, 42]. Despite this, few studies have analyzed how different recreational activities may influence the tick-borne disease risk. In our study, most visitors spent several hours per week in green spaces, mainly in urban green spaces close to their residences, while some also visited other urban and rural green spaces in Stockholm County. At the Nacka nature reserve, visitors engaged in various activities, with walking along the paths being the most common. Still many also venture off trails, increasing the risk of tick encounters, especially for those without protective measures [29, 56]. Recommendations for preventing tick bites include avoiding tick habitats, wearing protective clothing, and performing tick checks after spending time in risky environments [71, 72]. Despite having a good understanding of ticks, a large discrepancy was found between our respondents’ knowledge about and their adoption of preventative behaviors. The Swedish public health agency advises vaccination against the viral infection TBE in southern Sweden. Although our study focuses on bacterial tick-borne pathogens, we included TBE vaccination as a protective measure in our interviews. Thirty-six percent of the visitors to the urban green space were vaccinated, but many were unsure if they had received booster shots according to recommendations. Regarding protective clothing, visitors often prioritized weather or activity-appropriate attire over tick protection. Repellents were used sporadically, often targeting mosquitoes rather than ticks. Some respondents were opting for alterative repellents such as coconut oil or lavender spray.

A core principle of the theoretical concepts underpinning many public health risk reduction programs is that knowledge of a health threat is important for adoption of risk mitigation [73]. Absence of vaccines against bacterial tick-borne infections makes personal protection measures recommended as the first line of defense against ticks. Personal protection measures have the benefit of incurring no or low costs but need consistent use to be effective [34]. Previous research found that men are less likely to use protective measures against ticks, consistent with the general knowledge of men’s greater risk-taking behavior compared to women [33]. In our study, we found no significant gender difference in protective behavior or any differences among age groups. Interestingly, most of the visitors with a history of tick-borne diseases were women. Earlier studies also show that women are affected more frequently than men by tick-borne diseases [74]. However, this may reflect a gender difference in health care seeking behavior [75]. Notably, visitors with a history of a tick-borne disease also spent time in green spaces several days per week and had multiple tick-bites every year. Therefore, innovative, and evidence-based approaches to improve education on ticks and tick-borne disease and to promote behavior changes in urban green spaces are urgently needed.

The wide range of activities performed in urban green spaces may explain some of the large variation in type of, and adherence to, tick bite prevention. Using the green space only for walking generally lead to a limited engagement in protective measures. A few visitors tried to avoid “typical tick habitats” such as “tall grass” or “shrubs” to stay away from ticks. However, we have in earlier studies shown that tick densities are higher in low vegetation [46]. Therefore, visitors avoiding tall vegetation will be likely to spend more time and make closer skin contact with low vegetation close to the trails increasing the risk for tick-borne diseases. In addition, most of the visitors only applied protective measures, such as wearing long sleeves, and tucking pants into socks, during specific recreational activities such as picking berries and mushrooms.

### Limitations

This study is both spatially and temporally restricted, only giving a snapshot of the dynamic system of interactions among ticks, hosts, and pathogens. However, even if we expect a short-term temporal variation, with year-to-year differences in both tick densities and pathogen prevalence, both microhabitat and habitat types will be fairly stable over a rather long period of time, especially in ecosystems where the management strategies aim to conserve specific ecological succession stages. We are using a standardized sampling technique which sometimes results in rather few ticks sampled. This set limitations on the possibilities to estimate the exact tick density and pathogen prevalence. Still, the results from this cross-sectional study show that it is possible to correlate crude tick densities and pathogen prevalence with microhabitat and habitat data in an urban green space and with population census data and visitor count data. The standardized sampling technique also allows us to make comparisons with other similar studies.

Even though we performed interviews at most sites, we cannot present individual visitor data at the scale of the different entry points. Instead, this data is used to discuss general patterns and individual actions in comparison to population census data, visitor numbers and tick hazard. In assessing human behavior and movement patterns in urban green spaces, we did not account for the visitor’s time spend or the frequency of contact between visitors and vegetation during their recreational activities. Our tick-borne diseases risk assessment in urban green spaces was intended as a test to integrate tick-borne disease hazard, with population exposure both as visitor numbers but also as human activities, behavior, and their prevention practices. Regarding prevention practices, we did not ask the visitors about their beliefs on prevention efficacy. Therefore, further research is needed to better understand the factors that are important for behavioral decision regarding human recreational activities in urban green spaces.

## Conclusions

The present study highlights the risk of tick-borne diseases in urban green spaces. Our findings can be used for information to the public and for urban green space planning and management. Public health risk estimations can also be useful in decisions regarding vaccination programs for tick-borne diseases. For the moment it is only possible to vaccinate against TBE, but there is ongoing development also for *Borrelia* vaccines. Even if there will be no public vaccination programs this information will still be important for individual decisions regarding vaccination against tick-borne diseases. The challenge for public health authorities is to promote increased awareness without causing alarm, so that the residents rather than avoiding outdoor recreational activities continue spending time in urban green spaces.

### Supplementary Information

**Supplementary Material 1. **

**Supplementary Material 2. **

**Supplementary Material 3. **

**Supplementary Material 4. **

**Supplementary Material 5. **

**Supplementary Material 6. **

**Supplementary Material 7. **

## Data Availability

Sequence data is provided within the supplementary information files. Other datasets used and/or analyzed during the current study are available from the corresponding author on reasonable request.

## Abbreviations

AIC: Akaike information criterion
BLAST: Basic Local Alignment Search Tool
cDNA: Complementary DNA
DNA: Deoxyribonucleic acid
GIS: Geographical Information System
GLM: Generalized Linear Model
GLMM: Generalized Linear Mixed Model
HGA: Human Granulocytic Anaplasmosis
LB: Lyme Borreliosis
PBS: Phosphate-buffered saline
PCR: Polymerase chain reaction
qPCR: Quantitative PCR or real-time PCR
SCB: Statistics Sweden
s.l.: Sensu lato
SMD: National Land Cover Database of Sweden
spp.: Species plural
s.s.: Sensu strictu
TBE: Tick-borne encephalitis
VIF: Variance inflation factor
