# Supplementary material for: Ticks - public health risks in urban green spaces
Source: BMC Public Health. 2024 Apr 13;24:1031. doi: 10.1186/s12889-024-18540-8 (PMC11015579; doi:10.1186/s12889-024-18540-8)
Supplement: Supplementary file 1 — Supplementary Material 1. [file 12889_2024_18540_MOESM1_ESM.docx]

**Additional file 1**. Detail information from each entry point.

The **Sickla** entry point is situated south of central Stockholm, in the most urbanized part of the reserve. This entry point is close to an old farm estate from the 1800^th^ century, with an old English park and traces of a dam facility. The habitat surrounding the farm is moist mixed coniferous forest with scattered oak and hazel patches. At the entry point **Björkhagen**, there is a lush birch tree forest. In **Kärrtorp** and **Bagarmossen**, the vegetation is dominated by mixed coniferous forest, with scattered oak and hazel dominated patches in moist areas around lakes and streams. At **Klisätra** entry point, the landscape is dominated by meadows and small fragments of partly open areas surrounded by hardwood forests. Even the most remote entry point of the reserve, **Hellas recreation center**, is easily reached by bus only 30 minutes from downtown Stockholm. This area is dominated by large open grasslands and scattered broadleaved trees. The surrounding habitats are dominated by mixed coniferous forests.
