# Supplementary material for: Ticks - public health risks in urban green spaces
Source: BMC Public Health. 2024 Apr 13;24:1031. doi: 10.1186/s12889-024-18540-8 (PMC11015579; doi:10.1186/s12889-024-18540-8)

**Additional file 2**. Mopping technique

Tick collections were performed with the mopping technique. The mopping technique entails a white flannel blanket measuring 0.7m × 0.7m attached to a regular floor mop. The floor mop consists of a 60 cm head and an adjustable handle. Unlike flagging, the mopping technique allows the user to walk behind the mop, and easily adjust it to different vegetation heights.


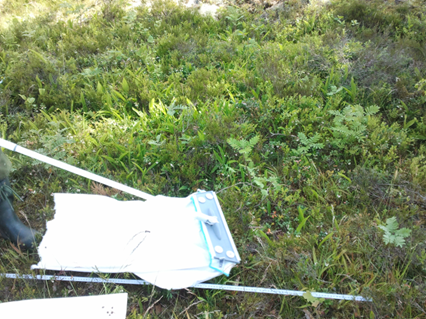

Supplement: Supplementary file 2 — Supplementary Material 2. [file 12889_2024_18540_MOESM2_ESM.docx]
