# Supplementary material for: Ticks - public health risks in urban green spaces
Source: BMC Public Health. 2024 Apr 13;24:1031. doi: 10.1186/s12889-024-18540-8 (PMC11015579; doi:10.1186/s12889-024-18540-8)
Supplement: Supplementary file 4 — Supplementary Material 4. [file 12889_2024_18540_MOESM4_ESM.docx]

| **Additional file 4**. Primers and probes used in pathogen screening and tick species determination. Abbreviations *ITS2* internal transcribed spacer, *gltA* bacterial citrate synthase gene, *16S* ribosomal RNA genes, *IGS* intergenic spacer region, *faB* fagellin B gene. | | | |
| --- | --- | --- | --- |
| **Primer/probe name** | **Target** | **Nucleotide sequence (5' → 3')** | **Amplicon size (bp)** |
| ApF | *A. phagocytophilum gltA* | TTTTGGGCGCTGAATACGAT | 64 |
| ApR |  | TCTCGAGGGAATGATCTAATAACGT |  |
| ApM |  | FAM-TGCCTGAACAAGTTATG-BHQ1 |  |
| Borrelia-F | *Borrelia spp. 16S rRNA* | GCT GAG TCA CGA AAG CGT AG | 131 |
| Borrelia-R |  | CAC TTA ACA CGT TAG CTT CGG TA |  |
| Borrelia-P |  | 6-FAM-CGC TGT AAA CGA TGC ACA CTT GGT-MGB |  |
| B5S-23S_F | *5S–23S rRNA IGS* | CTGCGAGTTCGCGGGAGA | 225-266 |
| B5S-23S_R |  | TCCTAGGCATTCACCATA |  |
| B5S-23S_Fn |  | GAGTTCGCGGGAGAGTAA |  |
| B5S-23S_Rn |  | TAGGCATTCACCATAGACTCTT |  |
| Bm_F | *Borrelia miyamotoi faB* | AGAAGGTGCTCAAGCAG | 156 |
| Bm_R |  | TCGATCTTTGAAAGTGACATAT |  |
| Bm_P |  | FAM-AGCACAACAGGAGGGAGTTCA AGC-BHQ1 |  |
| IXO-I2-F4 | *Ixodes sp. ITS2* | TCTCGTGGCGTTGATTTGC | 95 |
| IXO-I2-R4 |  | CTGACGGAAGGCTACGACG |  |
| Ipe-I2-P4 |  | FAM-TGCGTGGAAAGAAAACGA G-BHQ1 |  |
| Iri-I2-P4 |  | VIC-TGCTCGAAGGAGAGAACG A-BHQ1 |  |
