# Supplementary material for: Ticks - public health risks in urban green spaces
Source: BMC Public Health. 2024 Apr 13;24:1031. doi: 10.1186/s12889-024-18540-8 (PMC11015579; doi:10.1186/s12889-024-18540-8)
Supplement: Supplementary file 5 — Supplementary Material 5. [file 12889_2024_18540_MOESM5_ESM.docx]

Additional file 5. Survey questions

The questionnaire was developed to assess human behavioral aspects of tick-borne disease risks. It covered 4 thematic areas, including demographic and recreational risk factors, previous experiences with ticks and tick-borne diseases, usage of common recommended prevention behaviors, and knowledge of ticks and tick-borne diseases. Demographic and recreational behaviors were assessed to get an understanding of the overall behaviors and recreational activities in urban green spaces. In addition to age and gender, recreational risk factors were obtained by asking the visitors if they lived close to the nature reserve, how often they visit the green space and what activities they usually engaged in. The respondents were also asked about the frequency of tick bites and experiences of infections with tick-borne diseases. To determine the extent of preventative behaviors the visitors were asked whether they used insect repellents, other repellents, or engaged in any other behaviors to prevent tick bites. They were also asked if they dressed in a certain way while spending time in green spaces or when performing different types of recreational activities.

| **Area** | **Date** | **Interviewer** | | | | |
| --- | --- | --- | --- | --- | --- | --- |
| **Demographics** | | | | | | |
| Distance from residence to greenspace (meters) | Age | | Sex | | | |
| **Recreation** | | | | | | |
| How often do you visit greenspaces? |  | | | | | |
| Which greenspaces do you visit? |  | | | | | |
| Which activities do you engage in? |  | | | | | |
| Do you stay on the trails? |  | | | | | |
| Do you have a dog? |  | | | | | |
| Do you have any other pets? |  | | | | | |
| **Exposure to ticks and tick-borne diseases** | | | | | | |
| Have often do you have tick encounters? |  | | | | | |
| How many tick bites per year? |  | | | | | |
| Have you had a tick-borne disease? |  | | | | | |
| Geographical area of tick encounter? |  | | | | | |
| **Behavior** | | | | | | |
| Do you avoid ticks in nature? |  | | | | | |
| How do you usually dress when you are out in nature? |  | | | | | |
| Do you use any repellent? |  | | | | | |
| **Knowledge** | True | | | False | | Don’t know |
| If you get bitten by a tick is there is a large risk that you get ill? | **[ ]** | | **[ ]** | | **[ ]** | |
| Borrelia can be passed on from one person to another? | **[ ]** | | **[ ]** | | **[ ]** | |
| Mosquito repellent decrease the risk of getting a tick-bite? | **[ ]** | | **[ ]** | | **[ ]** | |
| There is a vaccine that can prevent TBE? | **[ ]** | | **[ ]** | | **[ ]** | |
| TBE can be treated with antibiotics? | **[ ]** | | **[ ]** | | **[ ]** | |
| Borrelia can be treated with antibiotics? | **[ ]** | | **[ ]** | | **[ ]** | |
| Which picture is showing a tick? | A) | | B) | | C) | |
