# Supplementary material for: Ticks - public health risks in urban green spaces
Source: BMC Public Health. 2024 Apr 13;24:1031. doi: 10.1186/s12889-024-18540-8 (PMC11015579; doi:10.1186/s12889-024-18540-8)
Supplement: Supplementary file 6 — Supplementary Material 6. [file 12889_2024_18540_MOESM6_ESM.docx]

**Additional file 6**. Risk of exposure to tick-borne pathogens

|  | Visitors | Ticks | Borrelia | Anaplasma | Sum | Risk |
| --- | --- | --- | --- | --- | --- | --- |
| Bagarmossen | 1 | 2 | 2 | 1 | 6 | Low |
| Björkhagen | 3 | 2 | 3 | 2 | 10 | High |
| Hellas | 1 | 1 | 3 | 3 | 8 | Moderate |
| Klisätra | 1 | 1 | 2 | 2 | 6 | Low |
| Kärrtorp | 2 | 2 | 2 | 3 | 9 | Moderate |
| Sickla | 3 | 1 | 2 | 3 | 9 | Moderate |
