# Supplementary material for: Ticks - public health risks in urban green spaces
Source: BMC Public Health. 2024 Apr 13;24:1031. doi: 10.1186/s12889-024-18540-8 (PMC11015579; doi:10.1186/s12889-024-18540-8)
Supplement: Supplementary file 7 — Supplementary Material 7. [file 12889_2024_18540_MOESM7_ESM.docx]

**Additional file 7. Aligned *Borrelia* nucleotide sequences based on PCR-products**

The identifier line, which begins with '>', gives the name of the **sample ID_ microorganism_gene target_developmental stage of tick**

**>N1_*Borrelia afzelii*_5S-23S ribosomal RNA intergenic spacer, partial sequence_*Ixodes ricinus*_nymph**

AGTTCGCGGGAGAGTAAGTTATTGCCAGGGTTTTTATTTTATACTTTAAATCTTGAGATTTATTTTTTAAATGTTTATATTATTTGAATGTATCTATTCAAATAATATAAAAAATAATATATATATTGACATGGATTAAACAAAGATATATATTATTTTATGTTGTATAAACCAAATGGCAAAATAGAGATGGAAGATAAAAATATGGTCTAAGTAATAAGAGTCTATGGTGAATGCC

**>N2_*Borrelia afzelii*_5S-23S ribosomal RNA intergenic spacer, partial sequence_*Ixodes ricinus*_nymph**

CGGGAGAGTAAGTTATTGCCAGGGTTTTTATTTTATACTTTAAACCTTGAATTTATTTTTTAAATGTTTATATTATTTGAATAAAACATTCAAATAATATAAAAAATAATATATATATTGACATGGATTAAACAAAGATATATATTATTTTATGTTGTATAAACTGACAGTAGGCAAAATAGAGATATAAGATAAAAATATGGTCAAAGTAATAAGAGTCTATGGTGAATGCC

**>N3_*Borrelia afzelii*_5S-23S ribosomal RNA intergenic spacer, partial sequence_*Ixodes ricinus*_nymph**

CTTCATCTCTATTTTGCCAATTTGTTTATACAACATAGAATAATATATATCTTTGTTTAATCCATGTCAATATATATATTATTTTTTATATTATTTGAATGTTTTATTCAAATAATATAAACATTTAAAAAATAAATTCAAGGTTTAAAGTATAAAATAAAAACCCTGGCAATAACTTACTCTCCCGCCAACTCTTCTG

**>N4_*Borrelia afzelii*_5S-23S ribosomal RNA intergenic spacer, partial sequence_*Ixodes ricinus*_nymph**

AGTTCGCGGGAGAGTAAGTTATTGCCAGGGTTTTTATTTTATACTTTAAATCTTCCCGGAATTTATTTTTTAAATGTTTATATTATTTGAATGTTTTATTCAAATAATATAAAAAATAATATATATATTGACATGGATTAAACAAAGATATATATTGTAGTACGTTGAATAGAAGCAAATGCCAGAATAGAAATG

**>N5_*Borrelia afzelii*_5S-23S ribosomal RNA intergenic spacer, partial sequence_*Ixodes ricinus*_nymph**

TCTTGGCTCACTCTTTTGCGAATTTTGTTTATACAACATAAAATAATATATATCTTTGTTTAATCCATGTCAATATATATATTATTTTTTATATTATTTGAGTATACCCTTCAAATGCTATCAACATTTAAAAATGGCATTCCCGAGGTGTAGTCTCGGGGGCGAAGACTGAGGCTAAGTTTTCCTCCCGCGGACTC

**>N6_*Borrelia afzelii*_5S-23S ribosomal RNA intergenic spacer, partial sequence_*Ixodes ricinus*_nymph**

TTCCTCTCTATTTTGCCTACTGTTAGTTTATAGCAACATAAAATAATATATATCTTTGTTTAATCCATGTCAATATATATATTATTTTTTATATTATTTGAATAAAACATTCAAATAATATAAACATTTAAAAAATAAATTCAAGATTTAAAGTATAAAATAAAAACCCTGGCAATAACTTACTCTCCCGCGAACTC

**>N7_*Borrelia afzelii*_5S-23S ribosomal RNA intergenic spacer, partial sequence_*Ixodes ricinus*_nymph**

TTCGCGGGAGAGTAAGTTATTGCCAGGGTTTTTATTTTATACTTTAAATCTTGAATTTATTTTTTAAATGTTTATATTATTTGAATGTTTTATTCAAATAATATAAAAAATAATATATATATTGACATGGATTAAACAAAGATATATATTATTCTATGTTGTATAAACAAATTGGCAAAATAGAGATGGAAGATAAAAATATGGTCAAACTGGTAAGAGTCTATGGTGGCTGCCT

**>N8_*Borrelia afzelii*_5S-23S ribosomal RNA intergenic spacer, partial sequence_*Ixodes ricinus*_nymph**

CGCGGGAGAGTAAGTTATTGCCAGGGTTTTTATTTTATACTTTAAATCTTGAATTTATTTTTTAAATGTTTATATTATTTGAATGTTTCATTCAAATAATATAAAAAATAATATATATATTGACATGGATTAAACAAAGATATATATTATTCTATGTTGTATAATACAAATTGGCAAAATAGAGATGGAAGA

**>N9_*Borrelia afzelii*_5S-23S ribosomal RNA intergenic spacer, partial sequence_*Ixodes ricinus*_nymph**

GTTCGCGGGAGAGTAAGTTATTGCCAGGGTTTTTATTTTATACTTTAAATCTTGAGATTTATTTTTTAAATGTTTATATTATTTGAATGTTTTATTCAAATAATATAAAAAATAATATATATATTGACATGGATTAAACAAAGATATATATTATTCTATGTTGTATAAACAAATTGGCAAAATAGAGATGGAAGATAAAAATATGGTCAAAGTAATAAGAGTCTATTGTGAATGACTA

**>N10_*Borrelia afzelii*_5S-23S ribosomal RNA intergenic spacer, partial sequence_*Ixodes ricinus*_adult male**

GAGTTCGCGGGAGAGTAAGTTATTGCCAGGGTTTTTATTTTATACTTTAAATCTTGAATTTATTTTTTAAATGTTTATATTATTTGAATGTTTTATTCAAATAATATAAAAAATAATATATATATTGACATGGATTAAACAAAGATATATATTATTCTATGTTGTATAAACAAATTGGCAAAATAGAGATGGAAGATAAAAATATGGTCAAAGTAATAAGAGTCTATGGTGAATGCCT

**>N11_*Borrelia afzelii*_5S-23S ribosomal RNA intergenic spacer, partial sequence_*Ixodes ricinus*_nymph**

CTTCCATCTCTATTTTGCCAATTTGTTTATACAACATAGAATAATATATATCTTTGTTTAATCCATGTCAATATATATATTATTTTTTATATTATTTGAATAAAACATTCAAATAATATAAACATTTAAAAAATAAATTCAAGGTTTAAAGTATAAAATAAAAACCCTGGCAATAACTTACTCTCCCGCGAACT

**>N12_*Borrelia afzelii*_5S-23S ribosomal RNA intergenic spacer, partial sequence_*Ixodes ricinus*_nymph**

GTCGGGAGAGGTAAGTTATTGCCAGGGTTTTTATTTTATACTTTAAATCTTGAGATTTATTTTTTAAATGTTTATATTATTTGAATGTTTTATTCAAATAATATAAAAAATAATATATATATTGACATGGATTAAACAAAGATATATATTATTCTATGTTGTATAAACAAATAGACAGTAGGCAACGGGAGAGATAAAAAT

**>N13_*Borrelia afzelii*_5S-23S ribosomal RNA intergenic spacer, partial sequence_*Ixodes ricinus*_nymph**

GTTCGCGGGAGAGTAAGCTATGGCCAGGGTTTTTATTTTATACTTTAAATCTTGAATTTATTTTTTAAATGTTTATATTATTTGAATAAAACATTCAAATAATATAAAAAATAAAATATATATTGACATGGATTAAACAAAGATATATATTATTTTATGTTGTATAAACAAATTGGCAAAATAGAGATGGAAGATAAAAATATGGTCAAAGTAATAAGAGTCTATGGTGAATGA

**>N14_*Borrelia afzelii*_5S-23S ribosomal RNA intergenic spacer, partial sequence_*Ixodes ricinus*_nymph**

TGTTCGCGGGAGAGTAAGTTATTGCCAGGGTTTTTATTTTATACTTCTAAATCTTGAATTTAGGTTTATTTAAATGTTCTATATYATTTGAATGTTTTATTCAAATGAATATAAAAAATAATATATATATTGACATGAGATTAAACAAAGATATATATTATTCTATGTTGTATAAACAAATTGGCAAAATAGAGAT

**>N15_*Borrelia burgdorferi* s.s._5S-23S ribosomal RNA intergenic spacer, partial sequence_*Ixodes ricinus*_nymph**

CTTCATCTCTATTTTGCCAATTTGTTTATACAACATAAAATAATATATATCTTTGTTTAATCCATGTCAATATATATTTTATTTTTTATGTTATTTGAATAACACATTCAAAAAATATAATATTTTAAAAAAGAAAAATAAAGTTTAAGTTTAAAGTATAAAAATAAAAACCCTGGTATACCCTACTCTCCT

**>N18_*Borrelia afzelii*_5S-23S ribosomal RNA intergenic spacer, partial sequence_*Ixodes ricinus*_nymph**

TCGGGGAGTTCGCGGGAGAGTAAGTTATTGCCAGGGTTTTTATTTTATACTTTAAATCTTGAATTCTATTTTTTAAATGTTTATATTATTTGAATGTTTTATTCAAATAATATAAAAAATAATATATATATTGACATGGATTAAACAAAGATATATATTATTCTATGTTGTATAAACAAATTGGCAAAATAGAGATGGAAC

**>N21_*Borrelia afzelii*_5S-23S ribosomal RNA intergenic spacer, partial sequence_*Ixodes ricinus*_nymph**

CTTCATCTCTATTTTGCCAATTTGTTTATACAACATAGAATAATATATATCTTTGTTTAATCCATGTCAATATATATATTATTTTTTATATTATTTGAATAAAACATTCAAATAATATAAACATTTAAAAAATAAATTCAAGATTTAAAGTATAAAATAAAAACCCTGGCAATAACTTACTCTCCCGCGAACTCCCAGG

**>N22_*Borrelia afzelii*_5S-23S ribosomal RNA intergenic spacer, partial sequence_*Ixodes ricinus*_nymph**

GTTCGCGGGAGAGTAAGTTATTGCCAGGGTTTTTATTTTATACTTTAAATCTTGAATTTATTTTTTAAATGTATTATATTATTTGAATGTTTTATTCAAATAATATAAACAAATAATATATATATTGACATGGATTAAACAAAGATATATATTATTCTATGTTGTATAAACAAATTGGCAAAATAGAGA

**>N23_*Borrelia afzelii*_5S-23S ribosomal RNA intergenic spacer, partial sequence_*Ixodes ricinus*_nymph**

CTGGGAGTTCGCGGGAGAGTAAGTTATTGCCAGGGTTTTTATTTTATACTTTAAATCTTGAGATTTATTTTTTAAATGTTTATATTATTTGAATGTTTTATTCAAATAATATAAAAAATAATATATATATTGACATGGATTAAACAAAGATATATATTATTCTATGTTGTATAAACAAATTGGCAAAATAGAGATGGAAGATAAAAATATGGTCAAAGTAATAAGAGTCTATGGTGAATGCCT

**>N26_*Borrelia afzelii*_5S-23S ribosomal RNA intergenic spacer, partial sequence_*Ixodes ricinus*_nymph**

CGGGAGAGTAAGTTATTGCCAGGGTTTTTATTTTATACTTTAAACCTTGAATTTATTTTTTAAATGTTTATATTATTTGAATGTTTTATTCAAATAATATAAAAAATAATATATATATTGACATGGATTAAACAAAGATATATATTATTCTATGTTGTATAAACAAATTGGCAAAATAGAGATGGAAGATAAAAATATGGTCAAAGTAATAAGAGTCTATGGTGAATGCCT

**>N27_*Borrelia afzelii*_5S-23S ribosomal RNA intergenic spacer, partial sequence_*Ixodes ricinus*_nymph**

TTATCTTCCGTCTCTATTTTGCCAATTTGTTTATACAACATAGAATAATATATATCTTTGTTTAATCCATGTCAATATATATATTATTTTTTATATTATTTGAATAAAACATTCAAATAATATAAACATTTAAAAAATAAATTCAAGATTTAAAGTATAAAATAAAAACCCTGGCAATAACTTACTCTCCCGCGAACTC

**>N28_*Borrelia afzelii*_5S-23S ribosomal RNA intergenic spacer, partial sequence_*Ixodes ricinus*_nymph**

AGTTCGCGGGAGAGTAAGTTATTGCCAGGGTTTTTATTTTATACTTTAAACCTTGAATTTATTTTTTAAATGTTTATATTATTTGAATGTTTTATTCAAATAATATAAAAAATAATATATATATTGACATGGATTAAACAAAGATATATATTATTCTATGTTGTATAAACAATGTAGGCAAAATAGAGATGGAAGATAAAAATATGGTCAAAGTAATAAGAGTCTATGGTGAATGCCT

**>N29_*Borrelia afzelii*_5S-23S ribosomal RNA intergenic spacer, partial sequence_*Ixodes ricinus*_nymph**

GTTCGCGGGAGAGTAAGTTATTGCCAGGGTTTTTATTTTATACTTTAAATCTTGAATTTATTTTTTAAATGTTCTATATTATTTGAATGTTTTATTCAAATAATATAAAAAATAATATATATATTGACATGGATTAAACAAAGATATATATTATTCCTATGTTGTATAAACAAATTGGCAAAATAGAGATGGAAGATAAAAATATGGTCAAAGTAATAAGAGTCTATGGTG

**>N30_*Borrelia afzelii*_5S-23S ribosomal RNA intergenic spacer, partial sequence_*Ixodes ricinus*_nymph**

GCGAACTCGCAGAATTTATCGGACAGAGGAAATAGATATAATTTCTTTGGTTTTAATTCACAGGTCAATATATCAATTATTTTTTATTTTATTTTCAATGAATCTTTCATTCATATAAGTGATTATCACCAATCGA

**>N31_*Borrelia afzelii*_5S-23S ribosomal RNA intergenic spacer, partial sequence_*Ixodes ricinus*_nymph**

TAGTTCGCGGGAGAGTAAGTTATTGCCAGGGTTTTTATTTTATACTTTAAACCTTGAAATTTATTTTTTAAGATGTTTATATTATTTGAATAAAACATTCAGAATAATATAAAAAATAATATATATATTGACATGGATTAAACAAAGATATATATTATTTTATGTTGTATAAACAAATTGGCAAAATAGAGATGGAAGATAAAAATATGGTCAAAGTAATAAGAGTCTATGGTGAATGCAT

**>N34_*Borrelia afzelii*_5S-23S ribosomal RNA intergenic spacer, partial sequence_*Ixodes ricinus*_nymph**

CTTCATCTCTATTTTGCCAATTTGTTTATACAACATAGAATAATATATATCTTTGTTTAATCCATGTCAATATATATATTATTTTTTATATTATTTGAATAAAACATTCAAATAATATAAACATTTAAAAAATAAATTCAAGATTTAAAGTATAAAATAAAAACCCTGGCAATAACTTACTCTCCCGCGAACT

**>N35_*Borrelia afzelii*_5S-23S ribosomal RNA intergenic spacer, partial sequence_*Ixodes ricinus*_nymph**

TCTTCCTCTCTATTTTGCCATTTGTTTATACAACATAGAATAATATATATCTTTGTTTAATCCATGTCAATATATATATTATTTTTTATATTATTTGAATAAAACCTTCAAATAATATCGGCCTTTAAAAAACGCATTCGCGATTTAAAGTATAAAATAAAAACCCTGGCAATACCTTACTCTCCCGCGAACTC

**>N36_*Borrelia bavariensis*_5S-23S ribosomal RNA intergenic spacer, partial sequence_*Ixodes ricinus*_nymph**

GGTCGGGGGAGAGGGGGGGATTGTCAGGATTTAGGAGAACACCGGAAGGGGGGGGGGGCGTTTTTAAAGGCATAAACGACTGTGAAGGTTTTATGGAAATAATATAAAAAATAATATATATATTGACATGGATTAAACAAAGATATATATTATTCTATGTTGTATAAACAAATGGCAAAATA

**>N37_*Borrelia afzelii*_5S-23S ribosomal RNA intergenic spacer, partial sequence_*Ixodes ricinus*_nymph**

TTCATCTCTATTTTGCCATTTGTTTATACAACATAAAATAATATATATCTTTGTTTAATCCATGTCAATATATATATTATTTTTTATATTATTTGAATGTTTTATTCAAATAATATAAACATTTAAAAAATAAATTTAAGGGTTTAAAGTATAAAATAAAAACCCTGGCAATAACTTACTCTCCCGCCACCCCGGGCACTCAGAGGAATT

**>N40_*Borrelia afzelii*_5S-23S ribosomal RNA intergenic spacer, partial sequence_*Ixodes ricinus*_adult female**

GTTCGCGGGAGAGTAAGTTATTGCCAGGGTTTTTATTTTATACTTTAAATCTTGAATTTATTTTTTAAATGTTTATATTATTTGAATGTTTTATTCAAATAATATAAAAAATAATATATATATTGACATGGATTAAACAAAGATATATATTATTCTATGTTGCTATAAACAATGAGGCAGCAGAGAGACGGACGATAAAAATATGGTCAAAGTAATAAGAGTCTATGGTGAATGCC

**>N41_*Borrelia garinii*_5S-23S ribosomal RNA intergenic spacer, partial sequence_*Ixodes ricinus*_nymph**

GTTCGCGGGAGAGTAAGTTATTGCCAGGGTTTTTATTTTATACTTTAAACATTGATTTTATTTTTTATGTTTTTAGATGTTCATGTTTTTGAATGTTTTATTCGAATAATATAAAAAATAAAATATATATTGACATGGATTAAACAAAGATATATATTATTCTATGTTGTATAAACAAATTGGCAAAATAGAGATGGAAGATAAAAATATGGTCAAAGTAATAAGAGTCTATGGTGAATGCC

**>N42_*Borrelia afzelii*_5S-23S ribosomal RNA intergenic spacer, partial sequence_*Ixodes ricinus*_nymph**

TGTTTGCGGGAGAGTAAGTTATTGCCAGGGTTTTTATTTTATACTTTAAATCTTGAATTTATTTTTTAAATGTTTATATTATTTGAATGTAATATTCAAATAATATAAAAAATAATATATATATTGACATGGATTAAACAAAGATATATATTATTCTATGTTGTATAAACAAATTGGCAAAATAGAGATGGAAGATAAAAATATGGTCAAAGTAATAAGAGTCTATGGTGAATGCCT
